# Vacuole inheritance regulates cell size and branching frequency of *Candida albicans* hyphae

**DOI:** 10.1111/j.1365-2958.2008.06545.x

**Published:** 2008-11-25

**Authors:** Veronica Veses, Andrea Richards, Neil A R Gow

**Affiliations:** The Aberdeen Fungal Group, School of Medical Sciences, Institute of Medical Sciences, University of AberdeenAberdeen AB25 2ZD, UK

## Abstract

Hyphal growth of *Candida albicans* is characterized by asymmetric cell divisions in which the subapical mother cell inherits most of the vacuolar space and becomes cell cycle arrested in G1, while the apical daughter cell acquires most of the cell cytoplasm and progresses through G1 into the next mitotic cell cycle. Consequently, branch formation in hyphal compartments is delayed until sufficient cytoplasm is synthesized to execute the G1 ‘START’ function. To test the hypothesis that this mode of vacuole inheritance determines cell cycle progression and therefore the branching of hyphae, eight tetracycline-regulated conditional mutants were constructed that were affected at different stages of the vacuole inheritance pathway. Under repressing conditions, *vac7*, *vac8* and *fab1* mutants generated mycelial compartments with more symmetrically distributed vacuoles and increased branching frequencies. Repression of *VAC1*, *VAM2* and *VAM3* resulted in sparsely branched hyphae, with large vacuoles and enlarged hyphal compartments. Therefore, during hyphal growth of *C. albicans* the cell cycle, growth and branch formation can be uncoupled, resulting in the investment of cytoplasm to support hyphal extension at the expense of hyphal branching.

## Introduction

*Candida albicans* is the most common agent of serious fungal infection in humans (Sandven, 2000; Almirante *et al*., 2005). Bloodstream infections caused by *C. albicans* have become common in patients recovering from surgical procedures and in those with immune dysfunction, and these infections have associated mortalities of between 30% and 40% – higher than that of most bacterial infections, including MRSA, *Clostridium difficile* and bacterial meningitis (Tortorano *et al*., 2004; Almirante *et al*., 2005).

*Candida albicans* is able to grow in three morphological forms: budding yeast, constricted pseudohyphal cells and parallel-sided true hyphae (Berman and Gow, 2004) and the ability to switch between different growth forms appears to be linked to its capacity to establish and disseminate infections in the human host (Lo *et al*., 1997; Gow *et al*., 2002; Saville *et al*., 2003; Zheng *et al*., 2004).

During true hypha growth, germ tubes of *C. albicans* emerge from the mother cell before the spindle pole is duplicated, before the chitin ring is formed at the neck, and before DNA synthesis occurs (Hazan *et al*., 2002; Sudbery *et al*., 2004; Crampin *et al*., 2005; Berman, 2006). Also, actin polarization leading to hyphal emergence occurs before START, suggesting that the evagination event is uncoupled from the cell cycle (Hazan *et al*., 2002). Germ tube extension rate is linear (Gow and Gooday, 1982a; Gow *et al*., 1986) – unlike the exponential extension rate of germ tubes of mycelial fungi (Trinci, 1971). Linear growth may occur because the biosynthetic resources for *C. albicans* germ tube growth do not increase as the hyphal cell elongates. During cell evagination, a relatively fixed volume of cytoplasm that is initially contained within the mother yeast cell migrates into the germ tube and a large vacuole is elaborated in the donor mother yeast compartment that replaces cytoplasm (Gow and Gooday, 1984; 1987). The apical compartment re-enters the cell cycle immediately after cytokinesis, but the vacuolated mother cell may not form a second germ tube for several cell cycles. This pattern of tip growth and subapical vacuolation is reiterated in all subsequent hyphal cell cycles so that the resulting mycelium is divided into discrete subapical compartments, most of which are extensively vacuolated and cell cycle arrested (Gow and Gooday, 1982b; 1987; Kron and Gow, 1995; Gow, 1997; Barelle *et al*., 2003; Barelle *et al*., 2006). These compartments then could form new germ tubes, hyphal branches or lateral yeast cells. Hyphae of *C. albicans* that are cultivated in nutrient-rich media have fewer vacuolated compartments and more branched compartments than hyphae that are grown in low nitrogen media (Barelle *et al*., 2003). The formation of vacuolated, G1-arrested, subapical compartments during hyphal growth may therefore reduce demands on protein synthesis for hyphal growth under nitrogen poor conditions.

The eukaryotic cell cycle is initiated at a stage when the cell achieves a critical cell size (Rupes, 2002). Size thresholds must also be achieved to progress through the G2/M mitotic checkpoint and for other aspects of cell growth (Jorgensen and Tyers, 2004; Barberis *et al*., 2007; Cook and Tyers, 2007; Jorgensen *et al*., 2007; Neuman and Nurse, 2007). It has been hypothesized that cell size measurement may reflect threshold concentrations of key regulatory proteins being achieved at specific stages of the cell cycle. We have hypothesized that vacuole compartment space is not relevant to these threshold cell sizes and therefore highly vacuolated cells can be regarded as being ‘smaller’ than other cells with the same overall cell volume (Barelle *et al*., 2003; 2006). The size of pseudohyphal cells has been estimated to be similar to that of budding cells if the vacuolar volume is subtracted from the overall cell size (Yokoyama and Takeo, 1983). The cyclin Cln3 controls both cell size and the initiation of cell division, but also regulates vacuolar biogenesis (Han *et al*., 2003). Therefore, vacuolation, growth and the cell cycle are linked both genetically and physiologically.

Vacuolar inheritance in *Saccharomyces cerevisiae* is a spatially and temporally ordered programme. The overall process of vacuolar inheritance is achieved by fragmentation of large vacuoles, and subsequent segregation of the smaller vesicles between the mother and daughter cells at cytokinesis. Finally, the fragmented vacuoles fuse to form a mature vacuole (Conradt *et al*., 1994). Vacuole fusion has been studied extensively *in vitro* and can be divided into priming, docking and fusion stages (Conradt *et al*., 1994; Wickner and Haas, 2000; Weisman, 2003). Several classes of mutant that affect vacuole inheritance (*vac*) have been isolated which result in distinct vacuole architectures and inheritance phenotypes. Vacuolar protein sorting (*vps*) mutants also affect vacuole partitioning and architecture (Wickner and Haas, 2000).

*Candida albicans* mutants with defects in vacuole inheritance have also been shown to have defects in hyphal growth and branching. *C. albicans vac8* mutants have been shown to have more symmetrically distributed vacuoles in hyphal compartments and an increased frequency of branching (Barelle *et al*., 2006). Null mutants in *ABG1*– a gene encoding a vacuolar protein of unknown function also exhibit a hyperbranched phenotype (Veses *et al*., 2005). A *C. albicans vps11* mutant failed to generate a vacuole compartment and was affected in hyphal morphogenesis, growth rate and macrophage killing (Palmer *et al*., 2003; 2005) and *C. albicans vsp34* mutants were affected in the timing of the cell cycle (Bruckmann *et al*., 2001). Mutants of *vac1* were affected in protein transport to the vacuole and exhibited multiple virulence defects (Franke *et al*., 2006) while *fab1* mutants were again defective in hypha induction but not virulence (Augsten *et al*., 2002). Therefore, numerous studies suggest links between vacuole dynamics and hyphal development in *C. albicans*.

To test the hypothesis that vacuole distribution in hyphal compartments of *C. albicans* affects cell cycle progression and hence branching frequency, we generated eight conditional mutants in the vacuole inheritance pathway. We show that under repressing conditions these mutants generated hyphae that had either highly vacuolated, sparsely branched compartments or more vacuole-depleted cells that branched more frequently. These observations suggest that cell cycle progression is affected directly by the pattern of vacuole inheritance, mediated by possible effects related to the set points for size thresholds that are required for cell cycle progression.

## Results

### *Candida albicans* vacuole inheritance pathway

The molecular machinery regulating vacuolar inheritance is not conserved across all fungi; however, blast analyses indicated a high degree of conservation in genes involved in the process of vacuolar inheritance between *S. cerevisiae* and *C. albicans* (Supporting Information, Table S1). Only the vacuole-specific Myo2p receptor, Vac17p, involved in co-ordination of the vacuolar inheritance within the cell cycle in *S. cerevisiae* (Ishikawa *et al*., 2003), is apparently absent in *C. albicans*, although *VAC8* which encodes the vacuolar receptor for Vac17p is present. The putative homologue of *VAM3* still remains to be formally annotated and is listed as IPF 1834 in the *C. albicans* database (http://genolist.pasteur.fr/CandidaDB/). In *S. cerevisiae* vacuole inheritance involves: (i) segregation of vacuolar vesicles originating from a fragmented mother vacuole, (ii) movement of the vesicles into the daughter cell and (iii) vacuolar homotypic fusion, involving proteins of the homotypic fusion and vacuole protein sorting complex (HOPS complex), proteins from the phosphatidylinositol 3-phosphate 5-kinase pathway, and components of the SNARE complex involved in vacuole fusion. The eight genes selected for mutagenesis represented examples of genes involved in each of these steps –*VAC1*, *VAC7*, *VAC8*, *FAB1*, *VAM3*, *YKT6*, *VAM2*(*VPS41*) and *VAM9* (*VPS16*). *VAC1* encodes a vesicle transport protein with roles in endosomal transport, metal ion homeostasis, virulence, adhesion, hyphal growth and chlamydospore formation (Franke *et al*., 2006). The *vac1* mutant of *S. cerevisiae* is defective in the vacuolar segregation process (Weisman and Wickner, 1992). Vac8p is the vacuolar receptor for Vac17p, involved in vacuolar movement (Veit *et al*., 2001). In *S. cerevisiae VAC8* participates in cytoplasm-to-vacuole protein targeting, the formation of nucleus-vacuole junctions and vacuole–vacuole fusion (Tang *et al*., 2006). *FAB1* and *VAC7* encode the phosphatidylinositol 3-phosphate 5-kinase in the yeast and its regulator (Gary *et al*., 1998; 2002), and are both required for priming and docking for vacuole fusion (Mayer *et al*., 2000). *VAM2/VPS41* and *VAM9/VPS16* encode components of the HOPS complex (Nakamura *et al*., 1997; Seals *et al*., 2000), and *YKT6* and *VAM3* encode proteins of the vacuolar SNARE complex, required for vacuolar fusion (Wickner and Haas, 2000). In *S. cerevisiae YKT6* participates in retrograde transport to the *cis*-Golgi and in three different biosynthetic pathways to the vacuole and is an essential gene (McNew *et al*., 1997).

### Generation of vacuole inheritance conditional mutants

To test the hypothesis that the size and distribution of vacuoles in compartments of *C. albicans* hyphae regulates branch formation, eight conditional mutants were constructed in which a single functional allele of a vacuole inheritance gene was placed under the control of a Tet-regulated promoter. The tetracycline regulatable system was chosen to circumvent difficulties in the analysis of essential genes and to allow all mutants to be generated in the same genetic background. First, heterozygous strains for all the mutants were constructed using appropriate primers (Supporting Information, Table S2) for disruption using the *URA3* mini-blaster system (Wilson *et al*., 2000). After 5-fluoroorotic acid (FOA) selection the promoter of the second copy of the gene of interest was replaced by the tetracycline promoter. Correct disruption of the first copy and correct integration of the tet promoter was checked by diagnostic polymerase chain reaction (PCR) (data not shown) and the functionality and kinetics of the tet promoter regulation was checked by reverse transcription polymerase chain reaction (RT-PCR), using gene-specific primers and the *EFB1* primers as a positive control (Maneu *et al*., 2000) (Supporting Information, Fig. S1). Mutant strains were grown in YPD for 24 h and then inoculated at a low cell density (OD_600_ 0.1) in YPD supplemented with doxycycline. After 2 h under repressing conditions no expression of the target gene was detected in the *ykt6* mutant. Growth for 4 h in doxycycline was required for full repression of *VAC1*, *FAB1* and *VAM9* expression. After 6 h no mRNA from the target gene was detected in any of the mutants (Supporting Information, Fig. S1). For all phenotypic analyses, mutant strains were pretreated with doxycycline for 24 h. This had no effect on the growth or morphogenesis of the wild-type strain (data not shown).

Vacuolar function in the conditional mutants, under repressing conditions, was mostly normal by several criteria. Quinacrine staining has been used to characterize vacuolar acidification, which is a requirement for proteolysis in this organelle (Weisman *et al*., 1987). Five of the eight conditional mutants were normal for quinacrine staining, whereas *ykt6* and *vam9* were partially defective in staining and *fab1* cells showed reduced quinacrine staining (Supporting Information, Fig. S2). Carboxypeptidase Y (CPY) activity measured in culture supernatants was used to evaluate the possible mislocalization of vacuolar hydrolases. There was no significant difference in external CPY specific activity in any of the conditional mutants under repressing conditions other than *vam3* which showed a slight increased activity (*P* < 0.01) after prolonged incubation with the CPY substrate (data not shown). The vacuole is also involved in pH homeostasis in response to changes in environmental pH (Weisman, 2003). No differences in growth rate were observed when the mutants were grown in M199 medium buffered with HEPES and adjusted to pH 4 or 7.5 (data not shown). Other than *vam9* and *fab1* the sensitivity of mutant strains was not affected by a range of stresses (osmotic, oxidative, temperature, metal ion) that in part impact on vacuole function (Supporting Information, Fig. S3). Therefore, most of the vacuole inheritance mutants were not affected markedly in the normal physiological functions of the vacuole.

### Vacuole mutants are differentially inhibited in hypha morphogenesis

No differences between the strains were observed when yeast cells were grown under permissive conditions in the absence of doxycycline. Under repressing conditions there were no significant differences between the growth rates of the yeast phase of wild-type and the conditional mutant strains in YPD at 30°C, except for the *ykt6* mutant, which was not able to grow in the presence of doxycycline (data not shown). This confirmed that *YKT6* is essential for *C. albicans*, as it is in *S. cerevisiae*. All phenotype measurements were performed on mutants grown under repressing conditions after 24 h preincubation in doxycycline. However, because *CaYKT6* is essential and its expression was rapidly repressed by doxycycline, phenotypic analyses for this conditional mutant were carried out on cells immediately after doxycycline addition – without overnight doxycycline pretreatment.

Hyphal morphogenesis was studied in cells cultivated in liquid medium, on glass slides immersed in liquid medium and on solid medium. In liquid media all mutant strains filamented normally under non-repressing conditions, except for the *ykt6* mutant, which was unable to support hyphal growth even when one functional copy of the gene was expressed. Under repressing conditions, the *vac1, vam2, vam3, vac7, vac8* and *fab1* mutants were able to undergo a complete yeast-hypha transition after 60 min in liquid YPD serum although some minor alterations were observed in the time at which the first germ tubes emerged in these strains. However, *vam9 and ykt6* were not able to form hyphae in liquid serum medium but had a limited capacity to form buds before growth was finally arrested in the presence of doxycycline.

**Fig. 1:**
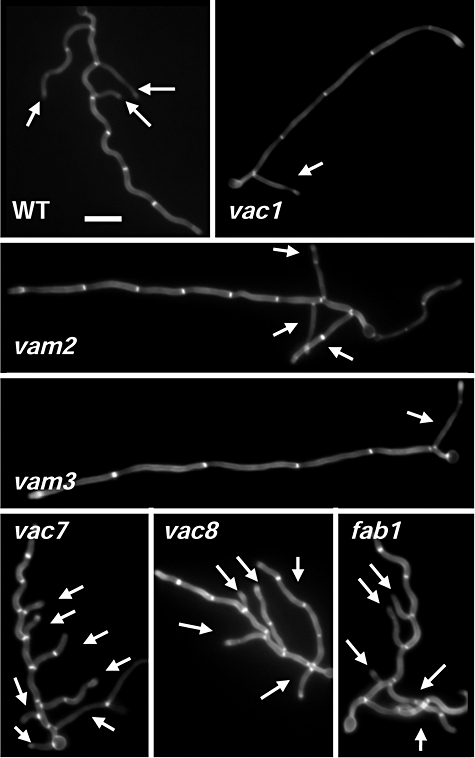
Hypha formation of tetracycline-regulated conditional vacuole mutants on glass slide cultures, under repressing conditions. Yeast cells were attached to poly l-lysine slides, induced to form hyphae and branches (arrows) using 1% (v/v) calf serum and stained with CFW. Scale bar represents 15 μm for all images.

For the filamentation positive mutants, branch formation could not be accurately measured in liquid cultures owing to the tendency of germ tubes to form aggregates. Therefore, hyphae were grown on poly l-lysine-treated glass slides in dishes containing liquid serum (Fig. 1). Under these conditions *vam9* and *ykt6* again did not form hyphae, but germ tube evagination, extension and branching (indicated by arrows in Fig. 1) occurred normally for the other mutants.

**Fig. 2:**
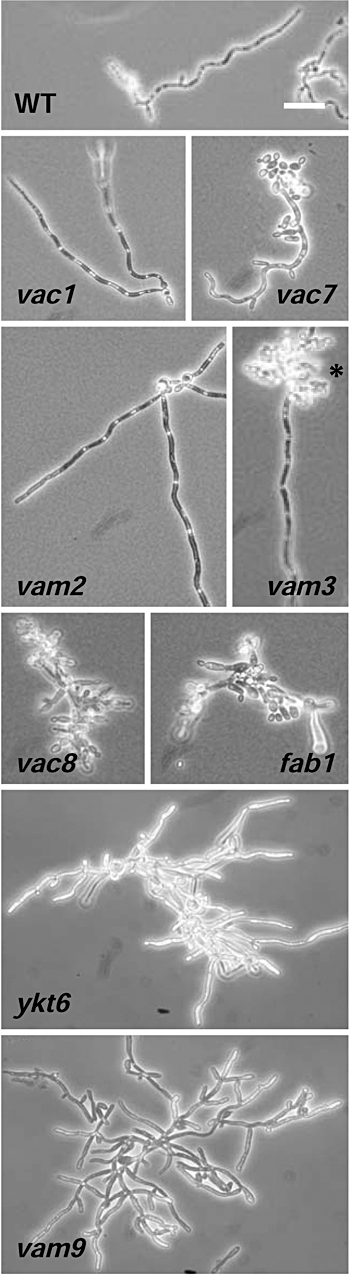
Phase-contrast images of hypha development of tetracycline-regulated conditional mutants after overnight growth on agar-slide cultures containing 1% (v/v) calf serum, under repressing conditions. *vac1*, *vam2* and *vam3* showed normal hyphal development. The *vac7*, *vac8, fab1, ykt6* and *vam9* mutants formed pseudohyphae under these conditions. The asterisk indicates yeast cells at the rear of the *vam3* hypha. Scale bar represents 20 μm for all images.

Hypha formation was also examined on solid media, using YPD supplemented with serum, ‘spider medium’ (Liu *et al*., 1994), and media containing *N*-acetyl-glucosamine as the sole carbon source (Supporting Information, Fig. S4). The vacuole mutants were compromised in their ability to form hyphae on a range of hypha-inducing agars. The *vam9* and *fab1* strains were unable to grow on Spider medium and *N*-acetyl-glucosamine. In YPD-serum agar, these strains formed small colonies whose edges were non-filamenting, as revealed by microscopical observation (data not shown). The *vac1*, *vam2*, *vam3*, vac7, *vac8* mutants generated normal wrinkly colonies, and microscopical inspection of the colonies revealed that the mutants grew predominantly as yeast cells and pseudohyphae on the solid media (data not shown). The *ykt6* mutant did not grow at all in plates supplemented with doxycycline. Therefore, its growth was analysed in the absence of doxycycline. *ykt6* formed normal wrinkly colonies in all media tested (Supplementary Fig. S4). Similarly, hypha formation was repressed for some of the mutants using agar slide-culture techniques. Normal hyphal development was observed in the *vac1*, *vam2* and *vam3* mutants (Fig. 2). However, the *vac8, vac7, fab1, ykt6* and *vam9* mutants formed pseudohyphae, even when the agar concentration was reduced to 1% (Fig. 2).

Therefore, vacuole mutants were deficient in filamentation on a range of hypha-inducing agars but formed normal non-constricted hyphae in liquid medium and when grown on glass slides that were immersed in liquid media containing calf serum (Fig. 1, arrows indicate branches) or *N*-acetyl-glucosamine (not shown).

### Vacuolar morphology of vacuole mutants in yeast and hyphal cells

**Fig. 3:**
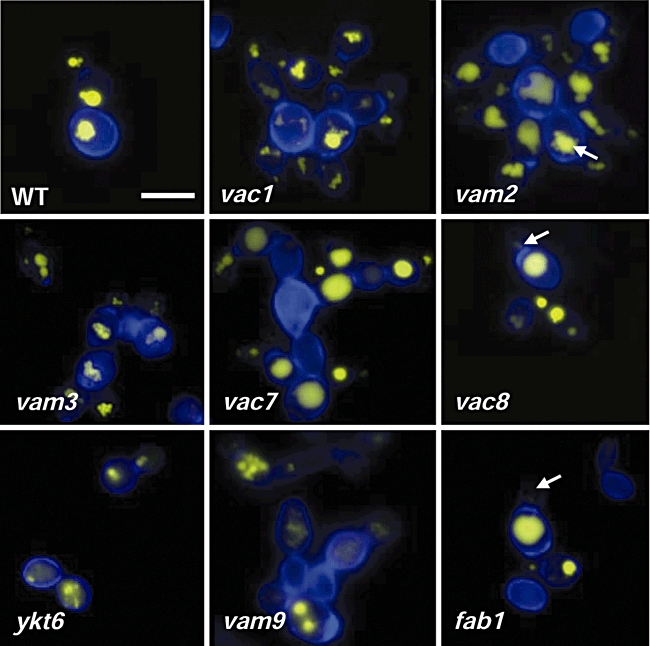
Vacuole morphology in budding cells of tetracycline regulated vacuole mutants. Yeast cells of CAI4-*URA3* and of the tetracycline-regulated mutants were stained with CDCFDA and CFW, under repressing conditions. The arrows indicate a multilobbed vacuole (*vam2*) and buds with no obvious vacuole (*vac8* and *fab1*). Scale represents bar 10 μm for all images.

The vacuolar morphology of the mutant strains was first defined in yeast cells, by staining the lumen of the vacuole with 5-(and 6-) carboxy-2′,7′-dichlorofluoresceindiacetate (CDCFDA). Cell walls were stained with Calcofluor White (CFW). One to three vacuoles, occupied approximately one-quarter of the area of a typical focal plane of a wild-type yeast cell (Fig. 3). To estimate the proportion of the cell that was represented by the vacuole in each cell, the number of pixels associated with the CDC-FDA fluorescence (vacuolar lumen) was divided by the total number of pixels contained inside the boundary determined by perimeter CFW fluorescence in each cell (see *Experimental procedures*).

In wild-type yeast cells the percentage of cell occupied by the vacuole was determined as 23 ± 1% (mean ± standard error; s.e.). In the *vac1* mutant highly fragmented vacuoles were observed, with a very prominent vacuole segregation structure. Few of the *vac1* cells had a vacuole in the bud, and the percentage of cell occupied by the vacuole was found to be similar with the wild-type strain (22 ± 1%; Fig. 3). Fragmented vacuoles were observed in the *vam2* and *vam3* mutants although *vam3* mutants had slightly smaller vacuoles and slightly reduced vacuole content compared with wild-type vacuoles, whereas *vam2* showed an enlarged, multilobbed vacuole (arrow, Fig. 3) that occupied 37 ± 2% of the cell. No other morphological defects were obvious in vacuole inheritance (Fig. 3). The *vac8* conditional and null mutant phenotypes appeared identical and were depleted in vacuoles in the emerging buds (Barelle *et al*., 2006). The *vac7* and *fab1* mutants shared this phenotype (cells lacking a vacuole indicated by the arrow in Fig. 3), although in these cases the mother cell vacuole space was enlarged – commensurate defective homotypic vacuole fission (43 ± 3; 37 ± 3, respectively, Fig. 3). Under repressing conditions for the *vam9* and *ykt6* mutants, CDCFDA stained diffusely throughout the yeast cell cytoplasm, although it was possible to detect a few intracellular compartments that resembled small vacuoles in some of the cells (Fig. 3). Therefore, the vacuole morphologies of the mutants were mostly consistent with orthologous mutants described in yeast cells of *S. cerevisiae*.

Hyphal cells of the vacuole mutants were stained with CDCFDA and CFW.

**Fig. 4:**
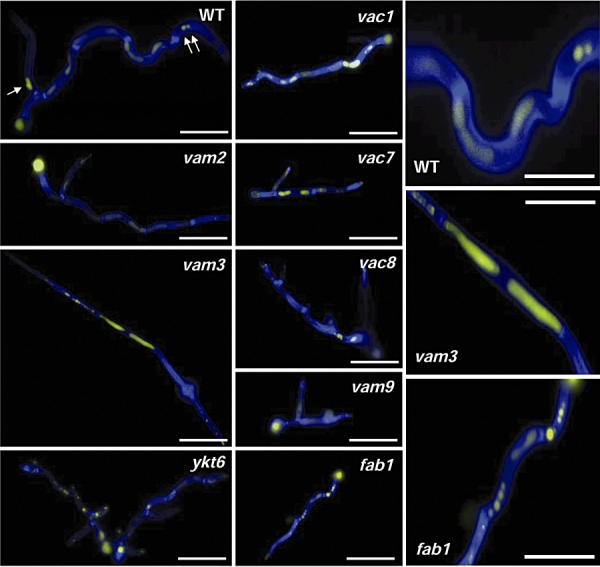
Vacuole morphology in hyphal cells of tetracycline regulated mutants. Vacuoles in hyphal compartments were stained with CDCFDA, under repressing conditions. CFW was used as a counter-stain to visualize the cell wall. The arrows indicate small vacuoles in the apical compartment of a hypha. Enlarged examples of wild-type (CAI4-URA3), enlarged (*vam3*) and smaller (*fab1*) vacuoles in subapical hyphal compartments are shown in the panels on the right. The scale bar is 10 μm for all images.

**Fig. 5:**
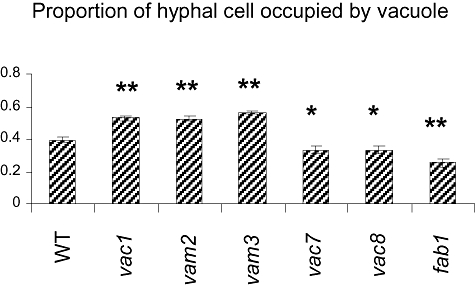
Proportion of the cell occupied by vacuole in subapical compartments of hyphal filaments. This ratio of vacuole: cytoplasm is calculated on the basis of the number of pixels representing the vacuole as a proportion of the number of pixels representing the whole cell, in a single, medial focal plane. Single stars indicate a *P*-value ≤ 0.05. For two stars, *P*-value is ≤ 0.01. Error bars represent standard errors.

In wild-type cells prominent vacuoles were present in subapical compartments as described previously (Barelle *et al*., 2003). In the growing apical compartment of the germ tube branches, smaller vacuoles were observed (Fig. 4, arrows). The vacuole morphology of the mutants grown under non-permissive conditions could be classified as enlarged, small or diffuse. Semi-quantitative analysis of space occupied by vacuole in the subapical compartments confirmed that the tet-regulated conditional mutants included examples of strains that had increased or decreased vacuolar cell volumes in the hyphal compartments (Fig. 5). The *vac1, vam2* and *vam3* mutants had increased vacuole content, with vacuolar spaces of 53 ± 7%, 53 ± 8% and 56 ± 8% (mean ± s.e.), compared with the wild type, 39 ± 7% (Figs 4 and 5). The *vac8*, *vac7* and *fab1* mutants had a decreased vacuolar space (33 ± 5%, 33 ± 5% and 25 ± 9%; Figs 4 and 5) and the vacuoles were smaller and mainly restricted to the more distal regions of the hyphae. The *vam9* mutant had a diffuse pattern of the CDCFDA staining, and it was not possible to visualize any structures which resembled normal vacuoles (Fig. 4). Prolonged culture of the *ykt6* mutant under repressing conditions led to progressive reduction in the size and number of vacuoles (Fig. 4). Therefore, in hyphal cells the *vac1*, *vam2* and *vam3* mutants tended to have increased vacuole content, while those of *vac7*, *vac8* and *fab1* had reduced vacuole content.

### Analysis of branching frequency

**Fig. 6:**
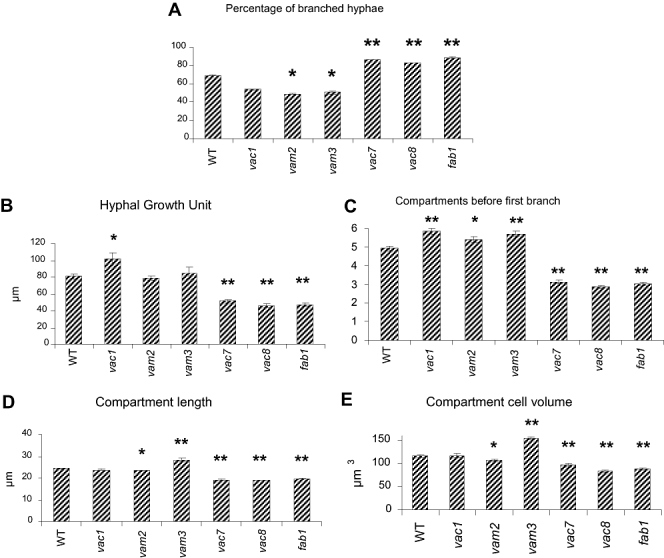
Analysis of branching frequencies, compartment lengths and cell volume in hyphal filaments of conditional mutants. A. Percentage of branched hyphae after 6 h of growth in 1% calf serum. B. HGU of mycelia for 12 h hyphal colonies grown attached to poly l-lysine slides for 12 h. C. Average number of compartments from the primary hyphal tip to the first branch assessed after 16 h of hyphal growth. D. The compartment length of subapical compartments measured as interseptum distances based on CFW-stained hyphal cells. E. Cell volumes of subapical compartments, calculated assuming compartments were of a constant diameter. Single stars indicate a *P*-value ≤ 0.05. For two stars, *P*-value is ≤ 0.01. Error bars represent standard errors.

Branching frequency was used as an indicator of the re-entry of hyphal cell compartments into the cell cycle. Branching frequency was quantified by measuring (i) the percentage of hyphal filaments that had one or more branches, (ii) the number of compartments from the apical cell to the first lateral branch and (iii) the Hyphal Growth Unit (HGU), defined as the total hyphal length in μm divided by the number of hyphal apices in an individual mycelium (Trinci, 1974). The lengths and volumes of the subapical compartments were also measured (Fig. 6).

In the absence of doxycycline no statistical differences were seen in branching frequency of the mutants compared with the control strain (*P* > 0.01), except for *vac1*, which showed decreased branching even under non-repressing conditions (data not shown). In most cases the *vac1*, *vam2* and *vam3* had decreased branching frequencies as was also evident in changes in hyphal growth unit and the numbers of compartments before the first branch (Fig. 6A–C). In contrast, *vac8*, *vac7* and *fab1* had increased branching frequencies according to all three measured parameters (Fig. 6A–C). The tendency to have a reduced branching frequency correlated with the presence of hyphal compartments with an increased vacuolar volume (Fig. 5). The corollary also applied – mutants that were hyperbranched had smaller vacuolar volumes (Fig. 5).

There were no large changes in the overall cell length of hyphal compartments; however, the average compartment lengths of the hyperbranched *vac8*, *vac7* and *fab1* mutants were approximately 20% shorter than wild type while compartments of the *vam3* were 15% longer of those in the wild type and controls (Fig. 6D). Some of the *vam3* cell compartments were as much as twice the length of those in the wild type and controls. Changes in cell volume reflected changes in cell length for all mutants (Fig. 6E). Therefore, vacuole inheritance mutants could form hyphae, but the branching frequency and hyphal compartment lengths and volumes were altered.

## Discussion

Filamentous fungi are specialist heterotrophic foragers, and the fungal vacuole plays important roles in their foraging lifestyle as well in terms of their roles in ion homeostasis and protein turnover and cellular homeostasis (Klionsky *et al*., 1990; Wickner and Haas, 2000; Veses *et al*., 2008). In many fungi the vacuole can occupy a substantial part of the overall cell volume and this can have consequences to other aspects of their growth – such as cell cycle control and hyphal branching. In this study we show that a range of *C. albicans* mutants that were affected in vacuole biogenesis and vacuole inheritance pathways have phenotypes that are reflected in the branching frequency of mycelia. It is argued that changes in vacuole volume may affect cell cycle regulation, perhaps by altering cell-size mediated functions.

Filamentous fungi do not undergo cell separation but the total length of the mycelium and the number of constituent branches in a mycelial colony increases exponentially (Trinci, 1974; Gow and Gooday, 1982b). Therefore, filamentous growth of fungi is hardwired into the same cell cycle circuitry that ensures balanced growth is maintained during the unicellular divisions of yeast cells. Also, some fungi uncouple cellular growth from the cell cycle so that hyphal tip extension occurs in the absence of a nuclear cycle. For example, hyphae of plant pathogens such as *Uromyces* and *Ustilago* species grow across the surface of plants in order to locate an infection site, but not to assimilate nutrients, which are usually scarce on plant surfaces (Hoch and Staples, 1983; Steinberg *et al*., 1998). These hyphae do not undergo cytoplasmic expansion on leaf surfaces – instead a fixed volume of migrating cytoplasm supports the expanding apical cell and growth occurs in the absence of a nuclear cycle. Hyphal growth therefore generates highly vacuolated distal compartments behind the tips that are virtually devoid of cytoplasm. This contrasts with hyphal growth of *C. albicans* where a nuclear cycle is maintained during the process of extensive hyphal vacuolation. The ability to uncouple cellular growth from the nuclear cell cycle is adaptive in as far as it enables a hypha to move, search and explore an environment without dissipating limited nutrient resources by undergoing branching. *Basidiobolus ranarum* also generates vacuolated, anucleate compartments, but also undergoes regular nuclear divisions and a branch is formed each time the cytoplasm in the hyphal apex doubles in volume (Robinow, 1963). Therefore, the ecology of different fungi has led to modification of their cell cycles to balance the biosynthetic demands of growth with the availability of nutrients. In achieving this some fungi increase cell volume by expanding vacuole volume rather than the cytoplasm. The study of vacuolation in fungi is therefore of broad relevance to the physiology of many fungal species.

Although the vacuole is essential for viability, mutants defective in this vacuole inheritance are still able to achieve partial segregation of vacuole during cell division and such mutants have similar growth rates to that of wild type under nutrient replete conditions (Weisman, 2003). However, in *S. cerevisiae* the *vam9* (*vps16*) mutant has severe phenotypic defects and *YKT6* is essential reflecting the involvement of these gene products in cellular processes that are not directly related to vacuole function such as intra-Golgi vesicle-transport activity and lysosome function (McNew *et al*., 1997). In this study the relationship between vacuolation, hyphal growth and branch formation of *C. albicans* was investigated exploring the hypothesis that organelle volume influences cell cycle progression by influencing the time at which critical cell-size dependent functions, such as re-entry into the cell cycle, are executed. To test the hypothesis that vacuolar volume is a direct determinant of cell cycle progression and branching in *C. albicans*, we generated a series of conditional mutants in the vacuole inheritance pathway and examined the effect of such mutations on the branching frequency and hyphal compartment (phenotypes summarized in Table 1). These mutants were not markedly compromised in vacuole physiology as determined by quinacrine staining, CPY localization and their pH stress responses. However, under repressing conditions all conditional vacuole inheritance mutants failed to form hyphae on solid media but could form hyphae in liquid medium and when grown on glass slides that were immersed in liquid medium. This phenotype was observed previously for *vps11* (Palmer *et al*., 2003; 2005), *vac1* (Franke *et al*., 2006) and *fab1* mutants (Augsten *et al*., 2002) of *C. albicans*. This suggests that vacuole biogenesis may be regulated by mechanisms that promote growth on solid media. Other effects of vacuolar mutations have been reported previously that affect virulence of *C. albicans* and these are presumed to be pleiotropic effects owing to mislocalization of hydrolytic enzymes or other virulence factors of the fungus (Palmer *et al*., 2003; Kumamoto and Vinces, 2005).

**Table 1:** Summary of results.

Gene	Protein	Stage^a^	Vacuole phenotype in yeast	Vacuole phenotype in hyphae	Vacuole function^b^	Branching frequency^c^	Hyphal growth unit	Compartments to 1st branch	Compartment length	Compartment volume
Wild type	N/A	N/A	One to three vacuoles, occupy approximately one-quarter of the total yeast cell volume	Prominent vacuoles present in subapical compartments, smaller vacuoles in growing apical compartments	++++	N/A	N/A	N/A	N/A	N/A
*VAC1*	Vacuole membrane protein	1	Highly fragmented vacuoles, very prominent vacuole segregation structure, only a few cells have a vacuole in the bud	Increased vacuole content, extensively vacuolated cell compartments	++++	↓	↑	∼	∼	∼
*VAM2*	Component of the HOPS complex	3	Highly fragmented, slightly enlarged, multilobbed vacuoles	Increased vacuole content, slightly swollen vacuoles	++++	↓	↑	∼	∼	∼
*VAM3*	SNARE protein	3	Highly fragmented, small vacuoles	Increased vacuole content, extensively vacuolated cell compartments	+−++	↓	↑	∼	↑	↑
*VAC7*	Regulator of phosphatidylinositol 3-phosphate 5-kinase	3	Depleted of vacuoles in emerging buds, mother cell vacuole enlarged	Decreased vacuole content, vacuoles smaller and mainly restricted to distal regions of the hyphae	++++	↑	↓	↓	↓	↓
*VAC8*	Vacuole membrane protein, receptor for Vac17	2	Depleted of vacuoles in emerging buds	Decreased vacuole content, vacuoles smaller and mainly restricted to distal regions of the hyphae	++++	↑	↓	↓	↓	↓
*VAM9*	Component of the HOPS complex	3	Vacuole material stained diffusely throughout the cytoplasm, no compartments resembling normal vacuoles	As in yeast	−++−	ND	ND	ND	ND	ND
*FAB1*	Encodes phosphatidylinositol 3-phosphate 5-kinase	3	Depleted of vacuoles in emerging buds, mother cell vacuole enlarged	Decreased vacuole content, vacuoles smaller and mainly restricted to distal regions of the hyphae	−++−	↑	↓	↓	↓	↓
*YKT6*	SNARE protein	3	Vacuole material stained diffusely throughout the cytoplasm, no compartments resembling normal vacuoles	As in yeast	−+++	ND	ND	ND	ND	ND

a Involvement in stage of vacuole inheritance: 1, segregation, 2, movement, 3, fusion.

b Four different parameters of vacuole functionality were tested; acidification, enzyme, pH, sensitivity: +, normal, –, abnormalities.

c Branching frequency and other measurements: ↑, increase, ↓, decrease, ∼, no difference.

ND, Not done as mutant was filamentation – defective under suppressing conditions.

**Fig. 7:**
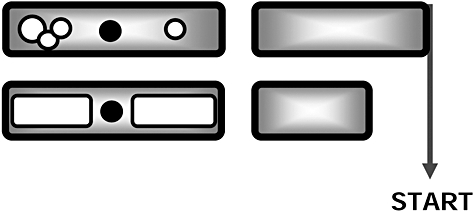
Model of how vacuolar volume influences cell size mediated, cell cycle regulation in subapical hyphal compartments of *C. albicans*. Extensively vacuolated cell compartments may be below the functional threshold cell size required to execute the Start function in G1. The two hyphal compartments on the left represent cells with different degrees of vacuolation: the upper cell being more extensively vacuolated than the lower cell. Drawn to the right of each of these hyphal compartment are corresponding cell compartments equivalent to the cytoplasmic volume alone (the total cell volume minus the vacuole volume). The functional cell volume in the upper cell is therefore large enough for the execution of the size-dependent Start function (arrow), while the lower, highly vacuolated cell, has insufficient cytoplasm for the execution of Start.

Measurements of hypha branching frequency demonstrated a correlation between the extent of vacuolation, and the progression of the cell cycle. Mutants with decreased vacuole content in hyphal compartments, including *vac7*, *vac8* and *fab1,* had higher branching frequencies compared with the control, and mutants with increased vacuolar contents such as *vam3* branched less frequently. Therefore, size of the vacuolar space correlated with the length of the cell cycle arrest in the subapical cells of the hyphae, suggesting that extensively vacuolated cell compartments may be below the threshold cell size required to execute the Start function in G1 (Fig. 7). The cell compartment length of *vac7*, *vac8* and *fab1* was decreased while that of *vam3* was increased relative to controls. Therefore, shorter compartments were found in mutants with smaller vacuoles and *vice versa*.

Recent work has established that the G1 cyclin Cln3p regulates both the initiation of START and vacuolar biology. In *S. cerevisiae* Cln3p binds to the scaffold signalling protein Bem1p and the rho–type Cdc42p GTPase that is essential for polarized growth (Han *et al*., 2005). Cln3 therefore regulates the length of the G1 in response to multiple environmental signals, and the absence of *CLN3* causes a cell and vacuolar size enlargement (Han *et al*., 2003). The *C. albicans CLN3* homologue is essential for viability, and its depletion in yeast cells result in a cell enlargement and the formation of highly vacuolated hyphae, even under conditions that would normally favour yeast growth (Bachewich and Whiteway, 2005; Chapa y Lazo *et al*., 2005). Little is known about the homeostatic mechanisms that control vacuolar volume in fungi; however, phosphatidylinositol 3,5-biphosphate has been shown to regulate the surface area to volume ratio of the vacuole by changing the number and size of the vacuole lobes, via vacuole fission (Bonangelino *et al*., 2002).

Both *S. cerevisiae* and *C. albicans* form pseudohyphal cells that are larger than the equivalent yeast cells. In *C. albicans* the volume of pseudohyphal cells has been estimated to be similar to that of yeast cells if the vacuolar volume is subtracted from the total cell volume (Yokoyama and Takeo, 1983) again suggesting that vacuole volume plays a role in cell cycle control. Numerous examples suggest an important role for cell extension and vacuolation. Some plants pathogens, such as *Uromyces* species and *Ustilago maydis*, exhibit vacuolar expansion during apical extension of hyphae (Hoch and Staples, 1983; Schuchardt *et al*., 2005) and in plant roots the increase in cell volume of cells in the zone of elongation behind the mitotically active apical meristem is due almost entirely to vacuole expansion. One benefit of vacuole expansion is to reduce the need for cytoplasmic biosynthesis, while permitting rapid cell extension, in particular under conditions of nutrient limitation.

Hyphae of *C. albicans* that have grown within infected tissues are often sparsely branched implying that conditions *in vivo* may be relatively nutrient poor (Barelle *et al*., 2003). The leading edge of sites of tissue invasion where host tissue is relatively intact may initially have less assimilatable low molecular weight nutrients available to the hyphal tip. In contrast, necrotic zones behind the infection margin which has become digested by the action of pathogen-derived secreted hydrolases may be richer in solutes, permitting re-entry of growth arrested compartments into the cell cycle via hyphal branching or lateral bud formation. Extensive vacuolation in *C. albicans* hyphal compartments enables continuous hyphal extension and septation to occur without branch formation at the end of each cell cycle. These observations also underline what is likely to be a general feature of the eukaryotic cell cycle – that cell size regulated checkpoints operates at the level of the cytoplasmic volume rather than total cell volume. The cell cycle paradigm in *C. albicans* also illustrates how the cell cycle of fungi can be subtly adapted to fit the ecological niche of the organism.

## Experimental procedures

### Strains, media and growth conditions

*Candida albicans* strains used in this study are listed in Table 2. Yeast cells were grown routinely in YPD (2% glucose, 1% yeast extract, 2% Bacto Peptone) or SD (0.67% yeast nitrogen base without amino acids, 2% glucose) media at 30°C with shaking. For growth of all mutants, media were supplemented with 25 μg ml^−1^ uridine as appropriate. For growth of conditional mutants under repressing conditions for the tetracycline promoter, strains were grown in YPD with 20 μg ml^−1^ doxycycline (Nakayama *et al*., 2000). To induce hyphal growth, cells were either inoculated on poly l-lysine-coated microscope slides containing 1% (v/v) newborn calf serum (Gibco) in water (Veses *et al*., 2005), on serum-agar slides as described previously (Barelle *et al*., 2003) or in liquid YPD containing 20% (v/v) newborn calf serum (Gow and Gooday, 1982b). Colony morphology was analysed on three types of solid hypha-inducing media: (i) YPD plus 20% newborn calf serum; (ii) spider medium (water, nutrient broth Oxoid, mannitol, K_2_HPO_4_, agar) (Liu *et al*., 1994); (iii) plates containing *N*-acetyl-glucosamine as a carbon source (yeast nitrogen base Difco, water, *N*-acetyl-glucosamine, agar) (Shepherd *et al*., 1980). A range of stress sensitivity assays were used to assess alterations in phenotype of the vacuole mutants (Kitanovic *et al*., 2005; Veses *et al*., 2005; Bates *et al*., 2006; Franke *et al*., 2006). Agar plates were supplemented with 12 mM caffeine; 1.5 mM NaCl; 2.5 M glycerol; 3 mM H_2_O_2_; 0.54 mM CaCl_2_; 0.005% sodium dodecyl sulphate, 60 μg ml^−1^ Congo Red, 15 μg ml^−1^ CFW; 10 mM MnCl_2_, or 10 mM CuSO_4_. To analyse the pH sensitivity of mutants, M199 medium was buffered with 150 mM HEPES and adjusted to pH 4 or pH 7.5. For phenotypic analyses strain CAI4-URA3 (NGY152) was used as a control.

**Table 2:** *C. albicans* strains used in this study.

Description or name	Strain	Genotype	Source or reference
CAI4-*URA3*	NGY 152	*ura3*Δ::λ*imm434*/*ura3*Δ::λ*imm434*, *RPS10*::*URA3*	Brand *et al*. (2004)
THE1		*ade2*::*hisG/ade2*::*his; ura3*Δ::λ*imm434*/*ura3*Δ::λ*imm434; ENO1/eno1*::*ENO1-tetR-ScHAP4ADxHA-ADE2*	Nakayama *et al*. (2000)
*vac1*	NGY 1139	THE1 *vac1*Δ::*Uradp1200/vac1*Δ*:URA3-TETp-VAC1*	This study
*vam2*	NGY 1140	THE1 *vam2*Δ::*Uradp1200/vam2*Δ*:URA3-TETp-VAM2*	This study
*vam3*	NGY 1141	THE1 *vam3*Δ::*Uradp1200/vam3*Δ*:URA3-TETp-VAM3*	This study
*vac7*	NGY 1142	THE1 *vac7*Δ::*Uradp1200/vac7*Δ*:URA3-TETp-VAC7*	This study
*vac8*	NGY 1143	THE1 *vac8*Δ::*Uradp1200/vac8*Δ*:URA3-TETp-VAC8*	This study
*vam9*	NGY 1144	THE1 *vam9*Δ::*Uradp1200/vam9*Δ*:URA3-TETp-VAM9*	This study
*fab1*	NGY 1145	THE1 *fab1*Δ::*Uradp1200/fab1*Δ*:URA3-TETp-FAB1*	This study
*ykt6*	NGY 1146	THE1 ykt6Δ::Uradp1200/ykt6Δ:URA3-TETp-YKT6	This study

### Strain construction

The tet-regulatable system (Nakayama *et al*., 2000) was used to construct conditional mutants affected in vacuolar processes. *C. albicans* was transformed by the lithium acetate procedure (Gietz *et al*., 1995), using primers are listed in Supporting Information Table S2. The first allele of each gene was disrupted using PCR-constructed disruption cassettes (Wilson *et al*., 2000) in the THE1 strain background that contains the TR transactivator (Nakayama *et al*., 2000). Ura^-^ segregants were selected on minimal medium containing uridine and 1 μg ml^−1^ FOA. The second copy of the target gene was placed under the control of the tetracycline promoter, using a cassette obtained by PCR amplification of p99CAU1 using primers that incorporated 20 bp homologous to the plasmid (p99CAU1) flanked with 60–80 bp of sequence of the target gene. RT-PCR analyses were carried out using Superscript II reverse transcriptase from Invitrogen, following manufacturer's instructions. Total RNA was isolated by mechanical disruption of the cells using a dismembrator, followed by use of the RNeasy Kit (Qiagen) according to manufacturer's instructions. Total RNA from cells grown with and without doxycycline were isolated for each mutant, reverse transcribed and PCR amplified using a specific set of primers for each gene, and *EFB1* primers as an internal control. All genes were found to be completely repressed after a period of 6 h.

### Vacuolar staining

Vacuoles in yeast and hyphal cells were visualized by costaining with CDCFDA (Molecular Probes Europe BV) and CFW (Sigma, UK). CDCFDA is a colourless compound that fluoresces once it reaches the vacuolar lumen and it is cleaved to its fluorescent form (Roberts *et al*., 1991). CFW is a fluorescent brightener that binds chitin, allowing the visualization of cell walls and septa (Pringle, 1991). Hyphal cells grown on poly l-lysine-coated microscope slides were incubated 5 h and then stained during the last 30 min with CDCFDA. Then cells were washed twice in water and stained with 1 mg ml^−1^ CFW solution. Vacuoles in mutant yeast cells were visualized by growing yeast cells to early log phase, harvesting, and resuspending the cells in 1 ml of YPD supplemented with CDCFDA (50 μM, final concentration). Cells were then incubated at 30°C for 30 min in the dark, harvested and resuspended in a 1 mg ml^−1^ CFW solution.

Acidification of the vacuole lumen was used as a measure of functionality. Cells were stained with quinacrine with minor modifications to published protocols (Weisman *et al*., 1987). Cells were grown to early log phase, harvested and resuspended in 1 ml of YPD buffered with 50 mM Na phosphate buffer supplemented with 200 μM quinacrine and incubated for 10 min, at 30°C in the dark. Cells were then harvested, and resuspended in 1 mg ml^−1^ CFW solution. Cells were observed using phase-contrast and fluorescence microscopy, performed using an Axioplan 2 microscope (Carl Zeiss, UK). CPY activity was used as an indicator of mislocalization of vacuolar hydrolases. The CPY-specific substrate *N*-benzoyl-L-tyrosine *p*-nitroanilide (NTPNA) was used to measure CPY activity in culture supernatants following published protocols with minor modifications (Palmer *et al*., 2003). NTPNA was diluted in a 1 M Tris-HCl, pH 7.5 at 0.5 mg ml^−1^ final concentration and 0.2 ml aliquots were added to the wells of microtitre plates, inoculated with 10^6^ yeast cells. Assays were performed in triplicate at 37°C measuring the absorbance of supernatants at 405 nm after 5 h.

### Measurement of hyphal branching and vacuole/cytosol ratio

Quantitative cell measurements of microscopical images were made using the Openlab version 5 software (Improvision, UK). Hyphal lengths and diameters were traced directly from electronic images and the branching frequency was measured by assessing (i) the average numbers of compartments from the hyphal apex to the first branch, (ii) the percentage of subapical hyphal compartments that had a branch or lateral bud and (iii) the HGU (Trinci, 1974). More than 150 individual hyphal filaments per strain were measured for (i) and (ii) above while > 10 colonies per strain were assessed for HGU analysis. Compartment length and volumes were measured in > 100 subapical cells of hyphal filaments. Volume was calculated assuming hyphal compartments were of constant diameter. The average diameters and the interseptum distances were determined from measurements of CFW-stained cells. The cellular space occupied by the vacuole was estimated by image analysis of con-focal microscopical images of yeast and hyphal cells. Volume measurements of small vacuoles often with complex geometry were difficult to assess directly from 2-D images or even from deconvolved 3-D images. Therefore, as an estimate of the proportion of the total cell accounted for by vacuole was obtained from measurements of the total area of medial optical sections of hyphal compartments that was represented by vacuole. As some smaller vacuoles were inevitably missed in single optical sections, this approach gave measurements that can be regarded as giving minimal estimates for the vacuole space – a parameter that is proportional to the true relative vacuolar volume. Vacuolar space was measured as the count of yellow pixels associated with CDC-FDA staining. The total cell volume was defined by the total number of pixels located inside the CWF-stained cell perimeter, using Adobe Photoshop® CS3. Only non-branched subapical compartments were used to determine the percentage of the cell occupied by vacuole. A minimum of 10 yeast cells or hyphal subapical compartments were analysed per strain.
