# Supplementary material for: Vacuole inheritance regulates cell size and branching frequency of Candida albicans hyphae
Source: Mol Microbiol. 2008 Nov 25;71(2):505–19. doi: 10.1111/j.1365-2958.2008.06545.x (PMC2680324; doi:10.1111/j.1365-2958.2008.06545.x)
Supplement: Supplementary file 1 [file mmi0071-0505-SD1.pdf]

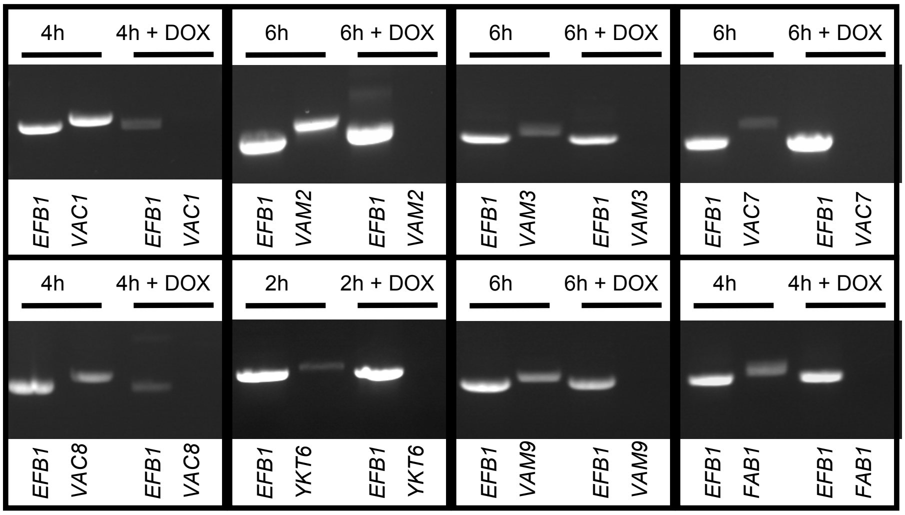

Supplementary Figure 1 Veses *et al*

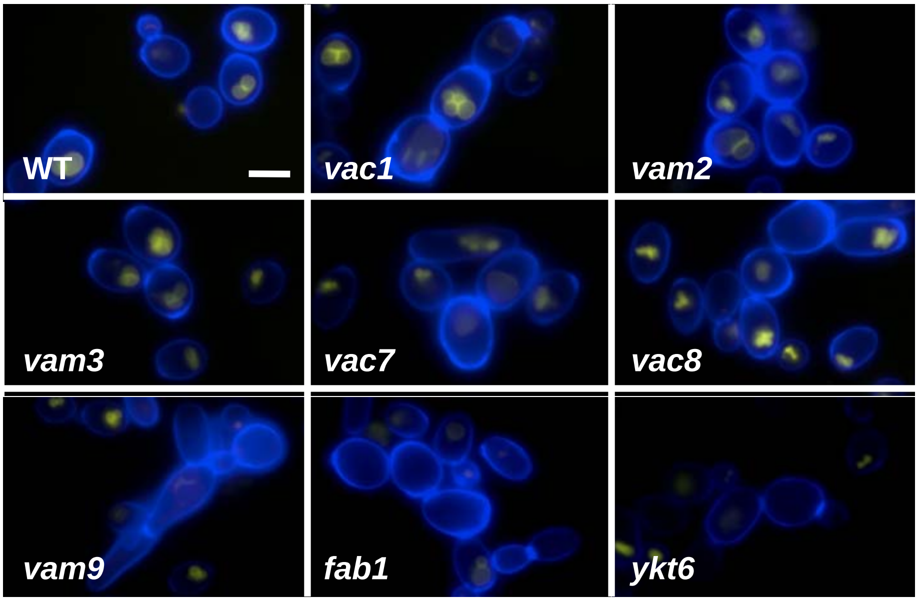

Supplementary Figure 2 Veses *et al*

A

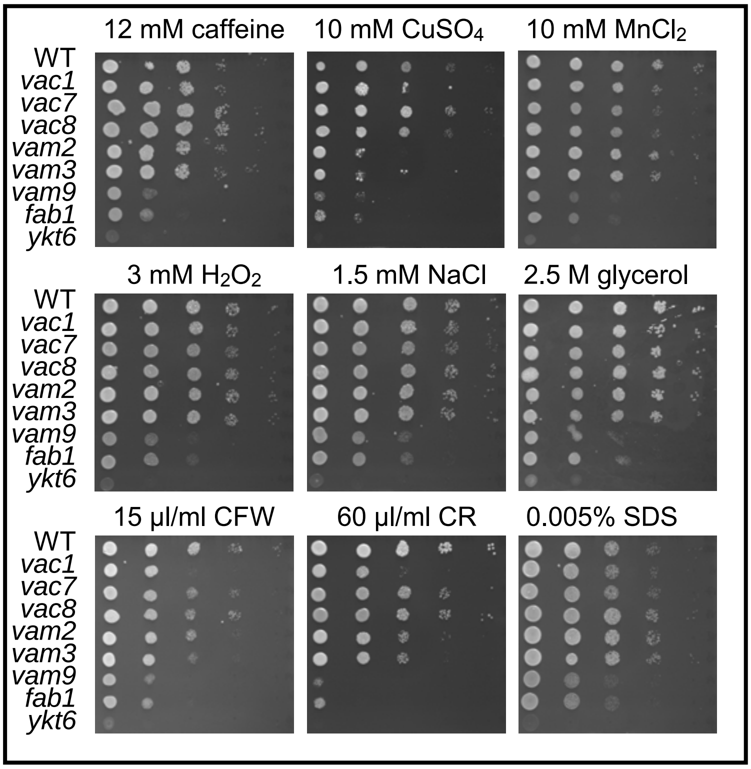

B

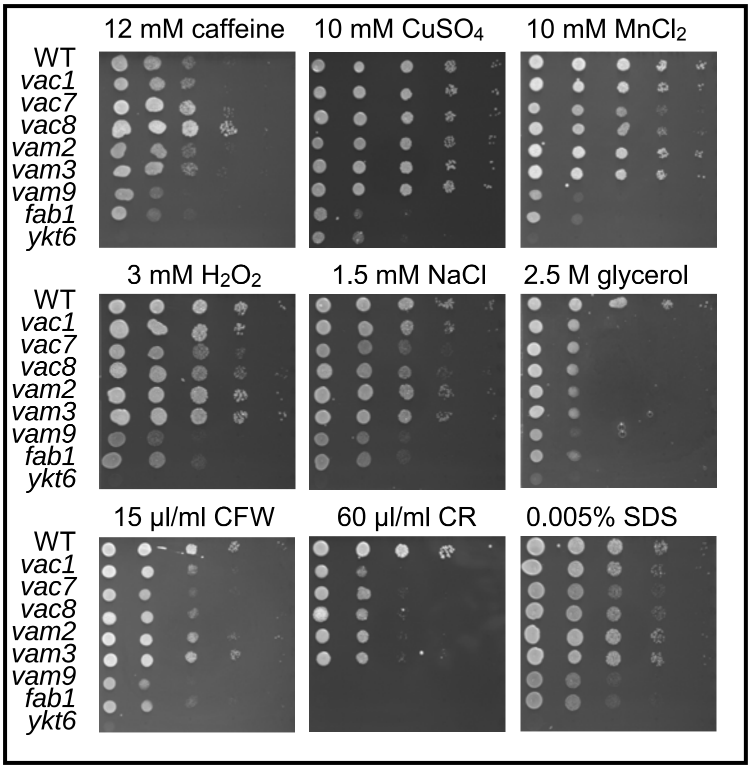

Supplementary Figure 3 Veses *et al*

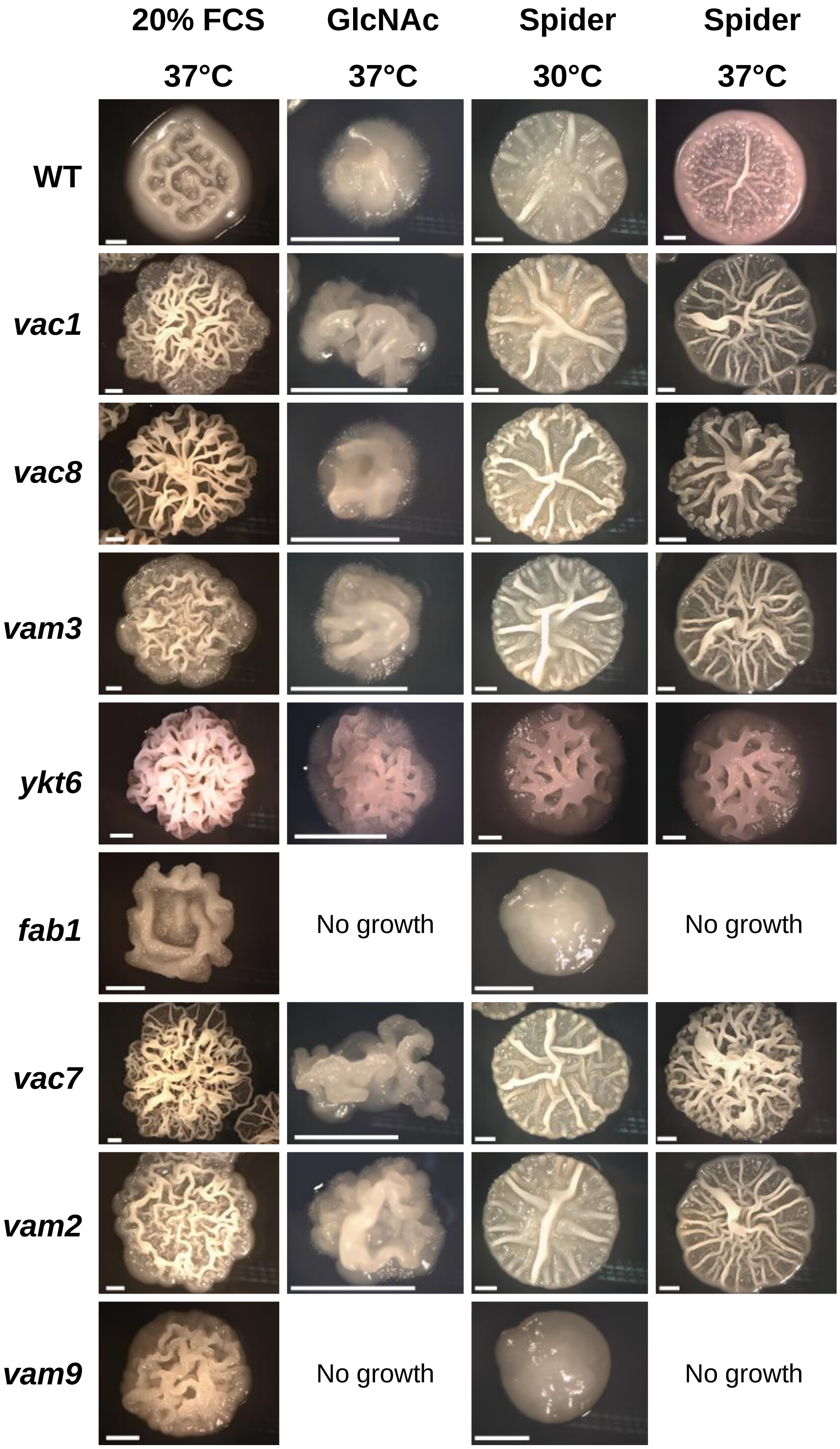

Supplementary Figure 4 Veses *et al*

Supplementary TABLE 1
Homology between *C. albicans* and *S. cerevisiae* vacuole genes

***C. albicans* gene** [1]	***S. cerevisiae* gene** [2]	**Percentage ID**[3]
VAC1/PEP7	*PEP7/VAC1*	28
VAM2/VPS41	*VPS41/VAM2*	32
VAM3(IPF1834, ORF19.5875)	*PEP12*	28
VAC7	*VAC7*	24
VAC8	*VAC8*	69
VAM9	*VPS16*	25
FAB1	*FAB1*	31
YKT6 (ORF19.2974)	*YKT6*	71

[1] Gene names for *C. albicans* were obtained from the Candida Genome Database (http://www.candidagenome.org)
[2] Gene names for *S. cerevisiae* were obtained from the Saccharomyces Genome Database (http://www.yeastgenome.org)
[3] The percentage of identity was obtained by using the BLASTP program (NCBI center), available at http://blast.ncbi.nlm.nih.gov/Blast.cgi

Supplementary TABLE 2 Primers used in this study

Primer	Sequence (5’-3’)
VAC1-1F $_{(1)}$	CGC CAC TAC AGG CCA CAT TGA TAT TTT CAT TAC ATG CAG ACC AGC AGC ACC CCA CCA AGA AAG ATC AAT CGG **TT TTC CCA GTC ACG ACG TT**
VAC1-1R $_{(1)}$	ACA TCT GCA CCA TAC TCC CCG AGA GTC ATA CTG TGG AGT CGT CTC ACC TTT ATC TGG ATT CCG TAA TCG TAC **TGT GGA ATT GTG AGC GGA TA**
VAC1-TF $_{(2)}$	CAG ATA ATG TGA TAC CCC AAG ATA TAG AAT TAT TTC TTA AAG ATC ATG AAG AAA CCA ACC AGT TGG ACG ATG TAG CCA **GTA ATA CGA CTC ACT ATA GGG**
VAC1-TR $_{(2)}$	CC AAT ACT AAA GCC TTG ATC CAG AAA TGA AAT CCG ATT GAT CTT TCT TGG TGG GGT GCT GCT GGT CTG CAT GTA ATG **CTA GTT TTC TGA GAT AAA GCT G**
VAC1-2F $_{(3)}$	ATG CAG ACC AGC AGC ACC CCA
VAC1-2R $_{(3)}$	GCC ATT CGG ATC ATC ACA AAC
VAC7-1F $_{(1)}$	TTC CTT TTT CTG CCA ACT TGA TCA AAT TCG ATG CTA CAT CCT AAT AAT TCA GTA GTA GTC GAT **TT TTC CCA GTC ACG ACG TT**
VAC7-RS $_{(1)}$	GAA TTG TTG CCT CAC TGG GTC ATG AAA ATA TTG AAG CTG TTC CCC CTC TAG TAT GGA TGG CGA TCG AAG **TGT GGA ATT GTG AGC GGA TA**
VAC7-TF $_{(2)}$	TCA CTA TCG TGA GGT AAA TAA CAA CTA CAG AGT TGT CAC AGT ATC CAA AAA AAA CTT TGT ACC TAT CAA TAC ATT TTA **GTA ATA CGA CTC ACT ATA GGG**
VAC7-TR $_{(2)}$	GG AGC ATC TGT ATT TTC ATT CAT GTT TCC AGT GCT AGA CAT ATC GAC TAC TGA ATT ATT AGG ATG TAG CAT CGA ATT **CTA GTT TTC TGA GAT AAA GCT G**
VAC7-2F $_{(3)}$	CGA TGC TAC ATC CTA ATA ATT CAG
VAC7-2R $_{(3)}$	CGA AGG TGA AGC AGT AAA CCT TTC
VAC8-1F $_{(1)}$	CAA GAA GAT ATC AAA ATG GGT GCC TGC TGT AGT TGT TTA GGT AAT CGT GGA GGT GAT GGA AGT CAT ACT CAG **TT TTC CCA GTC ACG ACG TT**
VAC8-1R $_{(1)}$	TTG CAC TTC GGA ATC AGC CAG TTG TAA CAA TAT CAA ATT TGG TTC CAA TAC ATC TCT ATT GTC TTC TCT AAC **TGT GGA ATT GTG AGC GGA TA**
VAC8-TF $_{(2)}$	GTT ACC TTC TAT TTA TTG TAC TAG TAT TTT ACA TTT GGG AAC ACC GTA ATA TCC TTA TCA ATA TTC TTG CGG GAT TAA **GTA ATA CGA CTC ACT ATA GGG**
VAC8-TR $_{(2)}$	TC TGC TAA TAA TAA CTG AGT ATG ACT TCC ATC ACC TCC AGC ATT ACC TAA ACA ACT ACA GCA GGC ACC CAT TTT GAT **CTA GTT TTC TGA GAT AAA GCT G**
VAC8-2F $_{(3)}$	ATG GGT GCC TGC TGT AGT TGT TTA
VAC8-2R $_{(3)}$	AAC TAA TTC TTG TCT ATT TTC ACC AGA
VAM2-1F $_{(1)}$	GGA TAG CAA CTA TTA TAA AAC ACG AAT TTT ATT AGT GGA GGT ATG GCT GGC CAG GTG ATT TAT TCT **TT TTC CCA GTC ACG ACG TT**
VAM2-1R $_{(1)}$	GTC ATC TTT GAA ACT TGC AAT TCC TGA TAT TAA ACT GTC TAA TTT GAA AAT ATG TTC AAC TTC CAC **TGT GGA ATT GTG AGC GGA TA**
VAM2-TF $_{(2)}$	TAC TAG ATT GAA TAA ATT ACC TGC TAA CTT TTT TGT TAA GGA CCC AGT CTC CAC CAG CAC TTT CAT GAA ACA GTA TTT **GTA ATA CGA CTC ACT ATA GGG**
VAM2-TR $_{(2)}$	CCC TGT TCC AAA ACA AAA TCT GAT CGT TTC CCC AAC CAA CCC TTG CTA GAA TAA ATC ACC TGG CCA GCC ATA CCT CC**C TAG TTT TCT GAG ATA AAG CTG**
VAM2-2F $_{(3)}$	ATG GCT GGC CAG GTG ATT TAT TCT
VAM2-2R $_{(3)}$	CAA CAT AAA ATC ATT CAA CCC TAA GTT
VAM3-1F $_{(1)}$	GAC TAA TCA TAT AAT GTC TTT TGC TAA TAT TGA TTT AGA GGC CCA AAA GGA ACC ATT ATT GAA AGG CAA **TT TTC CCA GTC ACG ACG TT**
VAM3-1R $_{(1)}$	AGT TTG TTC CTC TGT CTC TTT ACT TGG CCT AGT CAA TAA AGG TGT GGT TCT CTT TTT CTC TTG ATA CAC **TGT GGA ATT GTG AGC GGA TA**

Primer	Sequence (5'-3')
VAM3-TF (2)	TAG TTT CTT AAT CTT GCA AGA GAT TGA AAA GCC ATA CCA AAA ATT AGA ATT TCC ATT TAT CAA TCA CCA GCA TTA TAA **GTA ATA CGA CTC ACT ATA GGG**
VAM3-TR (2)	TTA GCA TCA TTA TCT TTG CCT TTC AAT AAT GGT TCC TTT TGG GCC TCT AAA TCA ATA TTA GCA AAA GAC ATT ATA TG**C TAG TTT TCT GAG ATA AAG CTG**
VAM3-2F (3)	ATG TCT TTT GCT AAT ATT GAT TTA
VAM3-2R (3)	TTC TAT TTC TCT GTT TCG TTC TTC GGT
VAM9-1F (1)	CCA ACC AAA TAA TCA ATG CCA TCT AAT CCA AGT TTT AAT TGG CTG AAG CTA CAA ATT GTA **TGT GGA ATT GTG AGC GGA**
VAM9-1R (1)	TTG GCT TAT GTT ATC CAT AGT GAC TAA ATC CTC GAC AAA GTT ATA CTC ATT GAA ACT ACC TAT **TT TTC CCA GTC ACG ACG TT**
VAM9-TF (2)	TTG TGA ATT ACG TAA CAC CGA TGA AAT CGA TGC CAC GTC TTT TGA AAT CAC AAA CTT GAA GTT ATC CGA GGA AAC TAA **GTA ATA CGA CTC ACT ATA GGG**
VAM9-TR (2)	TC GTA ACA TGT TCT GAT GTT ATA TAC ATT TTG TAG CTT CAG CCA ATT AAA ACT TGG ATT AGA TGG CAT TGA CTA TTA **CTA GTT TTC TGA GAT AAA GCT G**
VAM9-2F (3)	ATG CCA TCT AAT CCA AGT TTT AAT
VAM9-2R (3)	GTC TGA GGC AGA ATA GCT TGC
FAB1-1F (1)	AAC AAG ACC CTA CGA ATA TCT ACC ATG CAA TTC CCC CGT ATG CAA TCG ATG GCG AAT AGC ACC ACC **TGT GGA ATT GTG AGC GGA TA**
FAB1-1R (1)	AGC AAC AGA GTC ACC GTA TCT TGC ACT TAA TTC AAA TTG TAG AG ATT GTT CAT TTT AGA AAT AGA ACT CAA TT **TT CCC AGT CAC GAC GTT**
FAB1-TF2 (2)	TTG GAC AGG TGT ACA CTA CAA ACG TCT TGT TGT TGT ATC AAA TTT TGT GTA TGT GTT GTG AAT GGT TAT TGT TGT CTC CTT **GTA ATA CGA CTC ACT ATA GGG**
FAB1-TR2 (2)	CT TTC TAG TAG TGG TAA CTT GCG TTT TGA ATT GCT CAT GTC GTT AGG AGTATC TGG TGG CGA CCC AAT CAT AAT AAC **CTA GTT TTC TGA GAT AAA GCT G**
FAB1-2F (3)	ATG CAA TTC CCC CGT ATG CAA TCG
FAB1-2F (3)	AGA TGA TGC TGA TGA TGC GAC GGC
YKT6-1F (1)	GTA TAT TGA AAC AAA CTC AAA AGA TGA AGA TTT ATT ACA TTG GTA TTT TAA GAT CAA GTG GAG ACT **GTG GAA ATT GTG AGC GGA TA**
YKT6-1R (1)	ACC AAT ATA ATT ACC TTC TTC AAC ACT TTG TCT CTG TCC AGG TTG AGT TCT **TTT TCC CAG TCA CGA CTG TT**
YKT6-TF (2)	TGC TTA GCA ATT TAT CTT TGT TCC TAA AAC TCC ACA ACA TGG GCA AAC TCT TCA GCA TAG ACA CAG CTA AGC TAT TAT **GTA ATA CGA CTC ACT ATA GGG**
YKT6-TR (2)	TC TCT GGC TGA AGT TAA CTC TAA AGC CTT GTC TCC ACT TGA TCT TAA AAT ACC AAT GTA ATA AAT CTT CAT CTT TTG **CTA GTT TTC TGA GAT AAA GCT G**
YKT6-2F (3)	GAT GAA GAT TTA TTA CAT TGG TAT T
YKT6-2R (3)	TGA TCA CAT AAT CAA ACA ACA AGA ATT

Bold letters correspond to regions of homology to the cassette containing a selectable marker

(1) Primers used in the disruption of the first allele of each gene

(2) Primers used to place the second allele of each gene under the control of the tetracycline promoter

(3) Primers used for the diagnostic RT-PCR analysis
